# A population-based study of chronic hepatitis C in immigrants and non-immigrants in Quebec, Canada

**DOI:** 10.1186/s12879-017-2242-y

**Published:** 2017-02-13

**Authors:** Christina Greenaway, Laurent Azoulay, Robert Allard, Joseph Cox, Viet Anh Tran, Claire Nour Abou Chakra, Russ Steele, Marina Klein

**Affiliations:** 1 Division of Infectious Diseases, Jewish General Hospital, McGill University, 3755 Côte St. Catherine Road, Room E-0057, Montreal, PQ H3T 1E2 Canada; 2 Centre for Clinical Epidemiology, Lady Davis Research Institute for Medical Research, Jewish General Hospital, McGill University, Montreal, Canada; 3 Department of Oncology, McGill University, Montreal, Canada; 4 Montreal Public Health Department, Montreal, Canada; 5 Sexually Transmitted Infections Division, Montreal Public Health Department, Montreal, Canada; 6 Department of Mathematics and Statistics, McGill University, Montreal, Canada; 7 Division of Infectious Diseases, McGill University Health Center, McGill University, Montreal, Canada

**Keywords:** Hepatitis C, Immigrant, Incidence, Rates, Viral hepatitis

## Abstract

**Background:**

Immigrants originating from intermediate and high HCV prevalence countries may be at increased risk of exposure to hepatitis C infection (HCV) in their countries of origin, however they are not routinely screened after arrival in most low HCV prevalence host countries. We aimed to describe the epidemiology of HCV in immigrants compared to the Canadian born population.

**Methods:**

Using the reportable infectious disease database linked to the landed immigration database and several provincial administrative databases, we assembled a cohort of all reported cases of HCV in Quebec, Canada (1998–2008). Underlying co-morbidities were identified in the health services databases. Stratum specific rates of reported cases/100,000, rate ratios (RRs) and trends over the study period were estimated.

**Results:**

A total of 20,862 patients with HCV were identified, among whom 1922 (9.2%) were immigrants. Immigrants were older and diagnosed a mean of 9.8 ± 7 years after arrival. The Canadian born population was more likely to have behavior co-morbidities (problematic alcohol or drug use) and HIV co-infection. Immigrants from Sub-Saharan Africa, Asia and Eastern Europe had the highest HCV reported rates with RRs compared to non-immigrants ranging from 1.5 to 1.7. The age and sex adjusted rates decreased by 4.9% per year in non-immigrants but remained unchanged in immigrants. The proportion of HCV occurring in immigrants doubled over the study period from 5 to 11%.

**Conclusions:**

Immigrants from intermediate and high HCV prevalence countries are at increased risk for HCV and had a mean delay in diagnosis of almost 10 years after arrival suggesting that they may benefit from targeted HCV screening and earlier linkage to care.

**Electronic supplementary material:**

The online version of this article (doi:10.1186/s12879-017-2242-y) contains supplementary material, which is available to authorized users.

## Background

Health care utilization, costs and mortality due to hepatitis C infection are increasing in many low HCV prevalence countries in North America and Europe resulting in an enormous burden on society and health care systems [1–8]. Undetected chronic infections remain asymptomatic for decades until they progress to chronic liver disease and liver cancer [9]. HCV is now the leading infectious cause of death and the primary indication for liver transplants in Canada and the US [2, 3, 10, 11]. These sequelae are preventable through early screening and providing highly effective therapy that cures infection, stops the progression to liver disease and decreases all-cause mortality [12, 13]. Despite the benefits of curative therapy and recommendations for risk factor based screening almost half of all HCV infected persons in these low HCV prevalence countries remain undiagnosed [9, 14, 15]. Risk factor based programs recommend screening for those at increased risk for HCV exposure such as current or past intravenous drug use, those who have received blood products prior to routine HCV screening of the blood supply, hemodialysis, and incarceration [16–18]. Identifying all HCV infected individuals and successfully linking them to care is therefore an urgent priority to reverse the escalating burden of HCV.

Immigrants living in low HCV prevalence countries are a large underappreciated group that are disproportionately affected by HCV infection. Many were born in intermediate and high HCV prevalence countries and thus at increased risk to have been exposed to HCV in their countries of origin [19, 20]. In intermediate and high HCV prevalence countries HCV infection are acquired primarily iatrogenically through contaminated needles, medical procedures, or due to the receipt of unscreened contaminated blood products whereas in low HCV prevalence countries, the primary mode of transmission is through intravenous drug use (IDU) [16–18, 21]. Many immigrants from intermediate/high HCV prevalence countries will therefore be missed in current risk factor based screening programs. In certain low HCV prevalence countries such as Japan and Italy, the HCV epidemiology is more complex and reflects changing temporal patterns of HCV transmission with high HCV rates in older cohorts that were remotely infected through iatrogenic transmission (eg. blood transfusions and unsafe medical procedures), whereas recent infections are occurring in those with percutaneous exposure such as intravenous drug users, and/or among incarcerated individuals [9, 22, 23]. Recent estimates suggest that the foreign-born population has a higher HCV seroprevalence (~2%) compared to host populations and account for a substantial proportion of HCV cases (35–50% or more) in Canada and many European countries, even though immigrants make up less than 20% of the population [15, 19, 24]. Despite this, immigrants are not routinely screened for viral hepatitis before or after arrival in most host countries. In a population-based cohort, we aimed to determine the rates of newly reported HCV cases, the trend over time, and behavioral risk factors for exposure to HCV in immigrants as compared to the Canadian born population. The objective of this study was to identify immigrant groups who would benefit most from targeted HCV screening and early linkage to care. These data may ultimately be used to inform policy making and health care planning.

## Methods

We conducted a population-based retrospective study of all reported cases of hepatitis C and their associated health services in Quebec, Canada between January 1, 1998 and June 30, 2008 by linking three Quebec provincial administrative databases.

### Data sources

The Quebec public health mandatory reportable (notifiable) infectious disease (MADO) database in which all laboratory confirmed cases of HCV are reported using standard definitions since 1998 [25]. The majority of laboratories began reporting HCV in 1998 even though obligatory reporting of HCV came into effect in 2002. The *Régie de l’assurance maladie du Québec* (RAMQ), is the universal free health care plan in Quebec and covers over 99% of the Quebec born and landed immigrant population. RAMQ manages the following provincial administrative databases; 1) the Quebec hospital discharge database (MED-ECHO) in which discharge diagnoses are coded using the International Classification of Diseases, Ninth Revision (ICD-9) up until March 2006 and ICD-10 from April 2006 onward (ICD-10), 2) the physician reimbursement claims (RAMQ-PB) for inpatient and ambulatory medical services with visit diagnoses classified with ICD-9 codes and 3) the provincial prescription plan (RAMQ-Pharm) contains information on all dispensed medications and covers ~50% of the population. The Québec landed immigrant database (MIDI) contains demographic information on all immigrants and refugees (~1,258,000) who have arrived and were given permission to live in Quebec since 1985 [26].

### Cohort assembly

All cases of chronic HCV present in the MADO database between Jan 1, 1998 and June 30, 2008, were deterministically linked to the RAMQ databases through a unique health card number. All immigrants present in the MIDI database from January 1, 1985 to June 30, 2008 were deterministically linked to the RAMQ demographic database thorough the presence of a unique immigration number and allowed for classification of cases as occurring in immigrants (who arrived between 1985 and 2008) or in non-immigrants.

### Variables

All reported cases of chronic HCV present in the MADO database were included. Chronic HCV included those with either past or current (viremic) infection and were defined as the presence of HCV antibody (anti-HCV) detected by an enzyme immunoassay (EIA) confirmed by a second test (a different EIA, RIBA, INNO-LIA or PCR), or a very high level of anti-HCV antibodies not requiring a confirmatory test, or a positive HCV RNA by reverse transcriptase polymerase chain reaction (RT-PCR) [25]. Acute HCV cases were excluded. The MIDI database included information on country of origin, date of arrival, and immigrant class at time of landing. Countries of origin were grouped into regions of origin as classified by the World Bank (Additional file 1: Table A1) [27]. Immigration status at the time of acceptance as a permanent resident was classified as either a refugee or an immigrant. Hepatitis C behavioral risk factors including problematic drug use (both recreational and prescribed drugs) or underlying co-morbidities such as problematic alcohol use, alcohol-related liver disease, and HIV, occurring in the year prior to HCV diagnosis were identified in the MED-ECHO and RAMQ-PB databases through the presence of diagnoses classified by ICD-9/10 codes [28, 29]. Additional cases of drug use and HIV we identified through the use of methadone or HIV specific medications present in the RAMQ-Pharm database (Additional file 1: Table A2).

### Statistical analyses

Standard descriptive statistics were used when appropriate (*t*-test, Chi-2, and Fisher’s exact test). Rates of reported HCV cases/100,000 with 95% confidence intervals (CIs) for the entire study period and per year in immigrants and non-immigrants were estimated with a Poisson distribution. Stratum-specific rates by age, sex, region of origin, and immigration class for immigrants and by age and sex for non-immigrants were also estimated. Numerators were derived from the database produced in this study. The denominator was estimated using census data for the population of Quebec in 1996, 2001, and 2006, with linear interpolation for the intercensal years. Rate ratios (RRs) and 95% CI of overall and stratum specific rates of reported HCV cases in immigrants as compared to the Canadian-born population were calculated with Poisson regression. The age and sex adjusted trends of reported rates of HCV cases over the study period were modeled with Poisson regression in immigrants and non-immigrants, separately. To estimate the impact of unlinked cases [19.8% of all cases (*N* = 5255)] on the calculated rates and to determine if the linkage was differential for immigrants and non-immigrants, inverse probability weighting was used [30, 31]. Two separate models were constructed using age, sex, address, and year of diagnosis from complete cases as predictive variables for immigrant status. These variables were chosen as immigrants were older, less likely to be male and to live in different neighbourhoods compared to non-immigrants. Additional methods and results are shown in the Additional file 1: Table A3. All analyses were performed using SAS® 9.4 (SAS Institute Inc., Cary, NC, USA).

## Results

**Fig. 1:**
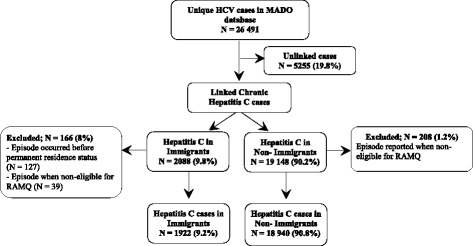
Flow chart for hepatitis C cases identification and Cohorts’ creation

Between 1998 and 2008, 26 491 unique cases of chronic HCV were identified in the MADO database. Of these, 20,862 cases were included in the final cohort; 1922 cases (9.2%) in immigrants and 18 940 cases (90.8%) in non-immigrants (Fig. 1). A total of 5255 (19.8%) cases did not link due to anonymous reporting by certain clinics or missing demographic or RAMQ numbers. Additional cases were excluded because episodes occurred during a period of ineligibility for RAMQ of > 6 months or prior to permanent resident status (immigrants). Unlinked cases (19.8%) were slightly younger (41 vs 43 years), more likely to be male and to reside in certain regions such as Montreal, than linked cases (Additional file 1: Table A4). They were not found to be differentially distributed between immigrants and non-immigrants after imputing with inverse probability weighting. The RR was similar for unweighted and weighted incidence; 0.85 (0.81–0.90) vs 0.87 (0.84–0.91) (Additional file 1: Table A3). All reported rates are therefore likely to be 20% higher than estimated for both groups.

Demographic characteristics and comorbidities of HCV cases are shown in Table 1. Cases in immigrants were older than in non-immigrants and were less likely to be male. Immigrant cases originated mainly from East Asia and the Pacific region (23%), Eastern Europe and Central Asia (14.7%), and the Middle East and North Africa (13%). The mean time to diagnosis of HCV after arrival in Quebec was 9.8 ± 7.0 years. There were significant differences between the prevalence of comorbidities. The Canadian born population was significantly more likely than immigrants to have behavioral co-morbidities such as problematic alcohol or drug use and to be HIV co-infected (Table 1). The prevalence of problematic drug use in immigrants was not significantly different by country/region of origin or immigration categories: the frequency of problematic drug use ranged between 1% in East Asia to 4% in the Middle East and North Africa, and was 2% overall for both refugees and other immigrants.

**Table 1:** Demographic characteristics of chronic hepatitis C cases (1998–2008) in immigrants and non-immigrants

Characteristics	Immigrants (*N* = 1922)	Non-immigrants (*N* = 18 940)	*P*-value
Age at time of HCV diagnosis (years)			
Mean (SD)	47.5 (15.1)	43.2 (13.3)	<0.001
Age group (years)			
< 15	11 (0.6)	104 (0.5)	<0.001
15–19	17 (0.9)	297 (1.6)	
20–29	133 (6.9)	2040 (10.8)	
30–39	452 (23.5)	5114 (27.0)	
40–49	588 (30.6)	6666 (35.2)	
50–59	324 (16.9)	2705 (14.3)	
60–69	193 (10.0)	1022 (5.4)	
≥ 70	203 (10.6)	991 (5.2)	
Sex			
Female	891 (46.4)	6004 (31.7)	<0.001
Male	1031 (53.6)	12,936 (68.3)	
Residence area by public health region			
Montréal	1488 (77.4)	7104 (37.5)	<0.001
Montérégie	157 (8.2)	2674 (14.1)	
Laval	105 (5.5)	747 (3.9)	
Capitale-Nationale	52 (2.7)	1713 (9.0)	
Laurentides	28 (1.5)	1582 (8.4)	
Outaouais	32 (1.7)	905 (4.8)	
Estrie	25 (1.3)	694 (3.7)	
Mauricie et du Centre-du-Québec	8 (0.4)	1132 (5.9)	
Other regions	35 (1.8)	2389 (12.6)	
Immigration category			
Permanent residents^a^	1209 (62.9)		
Refugee	522 (27.2)		
Missing	191 (9.9)		
Region of origin^b^			
East Asia and Pacific	443 (23.0)		
Eastern Europe and Central Asia	287 (14.9)		
Middle East and North Africa	255 (13.3)		
Latin America and Caribbean	205 (10.7)		
South Asia	181 (9.4)		
Western Europe	167 (8.7)		
Sub-Saharan Africa	152 (7.9)		
US, Australia, and New Zealand	22 (1.1)		
Missing	209 (10.9)		
Mean Time to diagnosis after arrival (years) (SD)	9.8 (7.0)		
Co-morbidities prior to diagnosis			
Problematic alcohol use	29 (1.5)	2419 (12.8)	<0.001
Alcohol-related liver disease	30 (1.6)	581 (3.1)	0.0003
Problematic drug abuse	47 (2.5)	4520 (23.9)	<0.001
HIV	23 (1.2)	874 (4.6)	<0.001

^a^Other categories included permanent residents who were workers, spouses, children, and students

^b^Countries of origin regrouped according to the World Bank criteria [27]. Top 10 countries were Vietnam (226), Pakistan (128), Cambodia (123), Romania (118), Haiti (111), Morocco (92), France (90), Egypt (60), Congo (55), and Russia (36) accounting for 54% of all cases

### Rates of reported cases of chronic hepatitis C

**Fig. 2:**
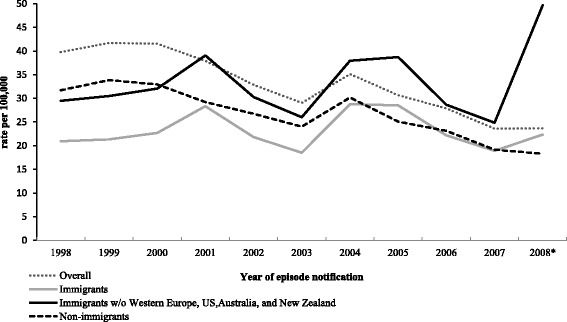
Reported rates of HCV Cases/100,000 overall and in immigrants and non-immigrants (1998–2008). Between 1998 and 2008, the rate of reported HCV cases adjusted for age and sex decreased by 4.89% per year in non-immigrants (95% CI = 4.85–4.93; *p* <0.001) compared to a 0.27% annual increase in immigrants (95% CI = 0.03–0.51; p = 0.028)

The rate of reported cases of HCV was higher in non-immigrants compared to all immigrants [27.1 vs 23.2/100,000, RR 0.85 (95% CI 0.80, 0.90)] (Table 2). Immigrants from low/intermediate income countries (ie. excluding immigrants from Western Europe, the US, Australia and New Zealand) however, had higher rates compared to non-immigrants; 32.3/100,000 with a RR of 1.19 (95% CI 1.13, 1.25). Rates of reported HCV cases adjusted for age and sex decreased by 4.89% per year in non-immigrants between 1998 and 2008 whereas it increased slightly by 0.27% per year in all immigrants (Fig. 2). The proportion of all HCV cases occurring in immigrants doubled during the study period; they accounted for 5.1% of all cases in 1998 and 11.1% of all cases in 2008. The overall reported rate of all HCV cases present in the MADO database (includes both linked and unlinked cases) between 1998 and 2008 was 33.9/100,000 (95% CI 33.5, 34.3). Supplemental data for all cases in the MADO database from 1991 to 2008 are presented in the Additional file 1: Table A5.

**Table 2:** Rates of Reported Cases of HCV per 100,000 from 1998 to 2008 (95% CI)

Characteristic	Immigrants		Non-immigrants		RR^a^ (95% CI)
	N	Reported rate (95% CI)	N	Reported rate (95% CI)	
Overall	1922	23.2 (22.1–24.2)	18 940	27.1 (26.7–27.5)	0.85 (0.8–0.9)
Low/Intermediate income source countries^b^	1733	32.3 (30.8–33.8)			1.2 (1.1–1.3)
Age groups (years)					
< 15	12	2.08 (0.90–3.26)	105	0.79 (0.64–0.94)	2.63 (1.45–4.77)
15–19	17	5.15 (2.70–7.60)	297	6.28 (5.56–6.99)	0.82 (0.50–1.34)
20–29	133	14.1 (11.7–16.5)	2040	22.6 (21.6–23.5)	0.62 (0.52–0.74)
30–39	452	28.4 (25.8–31.2)	5114	53.1 (51.7–54.6)	0.54 (0.49–0.59)
40–49	588	38.3 (35.2–41.4)	6666	56.9 (55.6–58.3)	0.67 (0.62–0.73)
50–59	324	24.2 (21.5–26.8)	2705	28.5 (27.5–29.6)	0.85 (0.75–0.95)
60–69	193	19.6 (16.8–22.4)	1022	16.5 (15.5–17.5)	1.19 (1.02–1.38)
≥ 70	203	20.5 (17.7–23.4)	991	17.1 (16.1–18.2)	1.20 (1.03–1.39)
Sex					
Female	891	21.1 (19.8–22.5)	60,004	17.0 (16.4–17.3)	1.25 (1.17–1.35)
Male	1031	25.3 (23.7–26.8)	12,936	37.8 (37.2–38.5)	0.67 (0.63–0.71)
Region of origin					
Sub-Saharan Africa	152	47.2 (39.7–54.7)			1.7 (1.5–2.0)
East Asia and Pacific	443	43.7 (39.6–47.8)			1.6 (1.5–1.8)
Eastern Europe and Central Asia	287	43.1 (38.1–48.1)			1.6 (1.4–1.8)
South Asia	181	41.5 (35.4–47.5)			1.5 (1.3–1.8)
Middle East and North Africa	255	19.0 (16.7–21.4)			0.7 (0.6–0.8)
Latin America and Caribbean	205	14.1 (12.2–16.0)			0.5 (0.5–0.6)
US, Australia, and New Zealand	22	7.8 (4.5–11.1)			0.3 (0.2–0.4)
Western Europe	167	6.6 (5.6–7.6)			0.2 (0.2–0.3)
Countries of origin^c^					
Viet Nam	226	53.0 (45.9–60.1)			1.9 (1.7–2.2)
Pakistan	128	123.1 (101.6–144.6)			4.5 (3.8–5.4)
Cambodia	123	116.6 (95.6–137.5)			4.3 (3.6–5.1)
Romania	118	39.3 (31.9–46.6)			1.4 (1.2–1.7)
Haiti	111	13.0 (10.5–15.4)			0.5 (0.4–0.6)
Morocco	92	24.0 (19.0–29.0)			0.9 (0.7–1.1)
France	90	10.0 (7.8–12.1)			0.4 (0.3–0.5)
Egypt	60	21.7 (15.2–27.2)			0.8 (0.6–1.0)
Congo	55	151.0 (110.0–192.0)			5.6 (4.3–7.3)
Russia	36	53.5 (40.7–66.3)			2.0 (1.4–2.7)

^a^Rate ratio comparing immigrants to non-immigrants, overall and in each category

^b^Immigrants from Western Europe, USA, Australia and New Zealand were excluded

^c^Top 10 Countries of Origin with the largest number of cases accounting for 54% of all immigrant cases

**Fig. 3:**
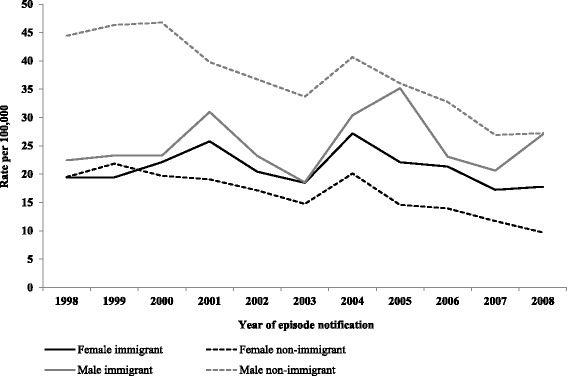
Reported rates of HCV cases/100,000 by sex and immigrant status (1998–2008). Between 1998 and 2008, the annual HCV cumulative incidence adjusted for age decreased by 5.21% per year in non-immigrant females and by 4.72% in non-immigrant males compared to 0.60% decrease in immigrant females and 1.18% annual increase in immigrant males (*p* <0.001)

**Fig. 4:**
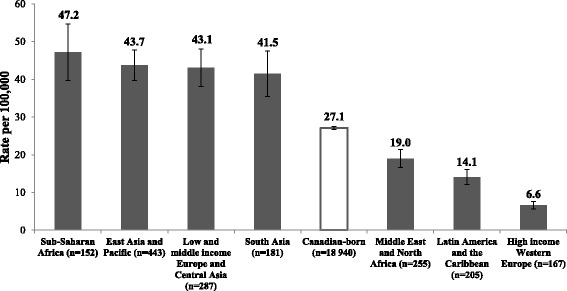
Reported rates of HCV cases /100,000 (95% CI) by region of origin

Reported rates of all hepatitis C cases per year stratified by sex are shown in Additional file 1: Figure A1. Reported HCV rates in all immigrant females were slightly higher over the study period compared to non-immigrant females with overall rates of 21.1 and 17/100,000 respectively [RR 1.25 (1.17–1.35)] (Table 2). HCV rates were lower in all immigrant males compared to non-immigrant males with overall rates of 25.3 and 37.8/100,000 respectively [RR 0.67; 95% CI 0.63–0.71]. Rates of HCV in non-immigrants males and females both decreased over the study period whereas HCV rates did not change in immigrant males and females (Fig. 3). Reported HCV rates also differed by age group between immigrants and non-immigrants; in immigrants cases were found in all age groups. Those who were younger (<15 years) and older (≥60 years) were more likely to be immigrants whereas young and middle aged adults (20–50 years) were more likely to be non-immigrants (Table 2). Reported HCV rates were higher in immigrants from Sub-Saharan Africa, East Asia and Pacific, Eastern Europe and Central Asia, and South Asia (47.2, 43.7, 43.1 and 41.5/100,000 respectively) compared to those in non-immigrants (27.1/100,000). Rate ratios ranged from 1.5 to 1.7 in these immigrant groups compared to non-immigrants (Table 2, Fig. 4). Reported HCV rates were particularly high in immigrants from Congo, Pakistan, Cambodia, Russia and Vietnam (Table 2).

## Discussion

Immigrants from intermediate and high HCV prevalence regions of Sub-Saharan Africa, Asia and Eastern Europe had a 1.5–1.7-fold higher risk of HCV compared to non-immigrants. Immigrants were older and less likely to be male compared to non-immigrants cases and were diagnosed a mean of almost 10 years after arrival. Non-immigrant cases were more likely than immigrant cases to have behavioral co-morbidities such as problematic alcohol or drug use, and to be HIV co-infected. The age and sex adjusted HCV annual notification rate decreased by almost 5% per year in non-immigrants but remained relatively unchanged in immigrants between 1998 and 2008. As a consequence, the proportion of HCV cases occurring in immigrants doubled over the study period from 5 to 11%.

In our study, immigrants from Sub-Saharan Africa, Asia and Eastern Europe had a higher HCV risk compared to non-immigrants whereas immigrants from North Africa and the Middle East and Latin America and the Caribbean had a lower risk. The pattern of the magnitude of HCV risk by region of origin in immigrants in our study reflects published epidemiology of HCV in immigrants after arrival in host countries as well as in their countries of origin [18, 24, 32]. HCV seroprevalence is a reasonable surrogate measure for the magnitude of HCV risk by region of origin and was used as there were no comparative studies on HCV notification rates in immigrants. HCV seroprevalence in Asia, Africa, and Central and Eastern Europe is intermediate to high (2 to 4%) whereas it is low (1–2%) in Canada, Western Europe, the US, Latin America and the Caribbean and most countries in North Africa except Egypt and Iraq [15, 19, 32]. Furthermore, high HCV reported rates in immigrants from Congo (151/100,000 and RR 5.6), Pakistan (123; 4.5), and Cambodia (117; 4.3) and are also consistent with global epidemiology as these countries all have a high HCV seroprevalence ranging from 2.3 to 6.7% [32].

We found major demographic and behavioral differences between immigrants and non-immigrants with HCV. The demographic and behavior characteristics of HCV in non-immigrants is consistent with the characteristics of HCV cases in Canadian surveillance data [15, 33]. HCV cases in Canada occur more frequently in males and in current and previous intravenous drug users and have a high risk of HIV co-infection [15, 33]. There is little data on HCV-infected immigrants in Canada or other high income countries. Our findings are similar to two other Canadian studies that also found that immigrants with HCV were older, and were less likely to be male or to have problematic drug use compared to non-immigrants [34, 35]. In our study there were significant differences in the HCV risk by age group for immigrants and non-immigrants. Male and female immigrants of all ages were found to have HCV, likely reflecting the fact that exposure to contaminated blood or unsafe medical procedures can occur at any age and equally by gender. In non-immigrants, cases were more likely to occur in young and middle age adults and reflects the age group at highest risk for initiation and active intravenous drug use [21]. Although the numbers are small, immigrants <15 years of age were more likely to have HCV than non-immigrants and may, in part, be due to perinatal transmission.

The decreasing trend of reported HCV cases seen in non-immigrants of 4.9% per year between 1998 and 2008 is consistent with the trends of notified HCV cases in Canada, the US and Australia over this same period [9, 33]. In contrast, rates of reported cases in immigrants during this same period remained unchanged. This could be explained by increased screening or reporting of HCV in immigrants, newly acquired infections after arrival, increased numbers of immigrants from at risk countries arriving over the study period, or a steady rate of imported HCV cases acquired in their country of origin in the setting of no routine post-arrival screening. The latter is the most likely explanation given that HCV screening in Canada during the study period was focussed on those with risk factors for HCV exposure such as intravenous drug use and did not identify immigrants as a high risk group [16, 36]. Furthermore, immigrants are at low risk for HCV transmission after arrival in Canada given the low prevalence of problematic drug use in this group and the fact that the number of new immigrants from at risk countries was stable during the study period [20]. With this dataset we cannot eliminate the possibility that immigrants may have become infected with HCV after arrival in Canada during periods of travel back to their countries of origin. Canadian immigrants are less likely to access health care due to several barriers including language, cultural difference or infrastructure [37, 38]. This coupled with the lack of a targeted HCV screening program may explain the 10-year timeframe to diagnosis after arrival. These findings, taken together, support the hypothesis that immigrants are most likely to have been infected with HCV in their countries of origin through unscreened blood products or unsafe medical procedures rather than through injecting drugs in Canada. The long asymptomatic period of chronic HCV and lower health care access may explain the long delay in diagnosis after arrival.

The major strengths of this study are that it is a large population based sample spanning almost a 10-year period in which immigration status was accurately assigned through linkage with the landed immigration database. All notified HCV cases were ascertained, were laboratory confirmed, and acute and chronic cases were identified and differentiated with standardized case definitions. This study has some limitations including the well-known underestimation of cases in a passive surveillance system such as MADO. These data are also influenced by health seeking behavior, non-nominal reporting for some groups, and increased screening of those at increased risk of hepatitis such as drug users. Due to the anonymous reporting of certain cases or due to missing demographic information or RAMQ numbers, almost 20% of cases did not link. Unlinked cases were not differentially distributed between immigrants and non-immigrants, thus calculated rates in this study are under estimated by 20% in both groups, but without misclassification between these groups. The increased reported HCV rate observed between 2002 and 2004 is likely due to the fact that HCV became reportable in Quebec in 2002 and that there was enhanced surveillance during this period of time [39]. We only have access to data up until 2008 however, we expect that the trends in case rates, underlying co-morbidities and time to diagnosis after arrival for immigrants found in our study would be similar to that after 2008. This is because there are no targeted HCV screening programs for immigrants in Quebec or Canada and this was the case both before and 2008. The accuracy of diagnostic codes to detect co-morbidities in RAMQ databases has not been validated, however good accuracy has been shown in other administrative databases [29]. Administrative coding for problematic drug use is limited by the fact that it is not possible to determine if drug use was intravenous or by another route and cases of prior drug use (in those not actively using drugs), may not be detected. We used the same codes for problematic drug use as were used in a recently published study [28]. Despite these limitations our data show important trends in HCV in immigrants compared to non-immigrants that are both plausible and are externally validated.

Untreated HCV is an enormous health and economic burden in Canada and other low HCV prevalence countries. This burden is projected to increase over the next decade unless the large pool of asymptomatic infected individuals are detected and treated prior to developing end stage liver disease [40, 41]. Eradication of HCV is now within reach given the recent availability of highly effective, well tolerated, short course, direct acting anti-viral treatments [12]. Identifying all asymptomatic HCV-infected individuals and successfully linking them to care is a critical issue that will need to be addressed in order to decrease the rising economic and individual burden from HCV.

## Conclusions

Immigrants born in intermediate and high HCV prevalence countries living in Canada are at increased risk for HCV, less likely to have behavioral risk factors for HCV and in the absence of a routine screening program were diagnosed a mean of almost 10 years after arrival. These immigrants are an important group to consider for targeted HCV screening and early linkage to care.

## Additional file

Additional file 1: Table A1. Worldbank classification for countries. **Table A2.** List of ICD-9/ICD-10 Codes used to identify co-morbidities. **Table A3.** Unweighted vs. Weighted Cumulative incidence of HCV cases per year (1998–2008). **Table A4.** Demographic Characteristics of Linked and Unlinked cases (1991–2008). **Table A5.** Annual Frequency and Rates of all Reported HCV Cases in the MADO database (1991–2008). **Figure A1.** Rates of all reported Chronic Hepatitis C cases per year stratified by sex. (DOCX 66 kb)

## Abbreviations

CI: Confidence interval
HCV: Hepatitis C virus
ICD: International classification of disease
IDU: Intravenous drug use
MADO: Maladies à declaration obligatoire; The Quebec public health mandatory reportable infectious disease
MED-ECHO: Maintenance et exploitation des données pour l’étude de la clientèle hospitalière; the Quebec hospital discharge database
MIDI: Ministère de l’Immigration, de la Diversité et de l’Inclusion; Provincial Ministry of immigration
RAMQ: The Régie de l’assurance maladie du Québec; Administers the provincial public health and prescription drug insurance plans
RAMQ-PB: RAMQ Physician billing; the physician reimbursement claims
RAMQ-Pharm: RAMQ Pharmacy billing
RR: Rate ratio
