# Supplementary material for: A population-based study of chronic hepatitis C in immigrants and non-immigrants in Quebec, Canada
Source: BMC Infect Dis. 2017 Feb 13;17:140. doi: 10.1186/s12879-017-2242-y (PMC5307836; doi:10.1186/s12879-017-2242-y)
Supplement: Additional file 1: Table A1. — Worldbank classification for countries. Table A2. List of ICD-9/ICD-10 Codes used to identify co-morbidities. Table A3. Unweighted vs. Weighted Cumulative incidence of HCV cases per year (1998–2008). Table A4. Demographic Characteristics of Linked and Unlinked cases (1991–2008). Table A5. Annual Frequency and Rates of all Reported HCV Cases in the MADO database (1991–2008). Figure A1. Rates of all reported Chronic Hepatitis C cases per year stratified by sex. (DOCX 66 kb) [file 12879_2017_2242_MOESM1_ESM.docx]

**A Population-based Study of Chronic Hepatitis C in Immigrants and Non-Immigrants in Quebec, Canada**

**Greenaway et al. 2016**

**ADDITIONAL FILE**

Table A1- Worldbank classification for countries

Table A2- List of ICD-9/ICD-10 Codes used to identify co-morbidities

Table A3- Unweighted vs. Weighted Cumulative incidence of HCV cases per year (1998-2008)

Table A4- Demographic Characteristics of Linked and Unlinked cases (1991-2008)

Table A5- Annual Frequency and Rates of all Reported HCV Cases in the MADO database (1991-2008)

Figure A1- Rates of all reported Chronic Hepatitis C cases per year stratified by sex

Table A1- Worldbank classification for countries

| **East Asia & Pacific** | **Sub-Saharan Africa** | **Latin America & Caribbean** | **South Asia** |
| --- | --- | --- | --- |
| American Samoa | [Angola](http://data.worldbank.org/country/angola) | Argentina | Afghanistan |
| [Cambodia](http://data.worldbank.org/country/cambodia) | [Benin](http://data.worldbank.org/country/benin) | [Belize](http://data.worldbank.org/country/belize) | [Bangladesh](http://data.worldbank.org/country/bangladesh) |
| [China](http://data.worldbank.org/country/china) | [Botswana](http://data.worldbank.org/country/botswana) | [Bolivia](http://data.worldbank.org/country/bolivia) | [Bhutan](http://data.worldbank.org/country/bhutan) |
| [Fiji](http://data.worldbank.org/country/fiji) | [Burkina Faso](http://data.worldbank.org/country/burkina-faso) | [Brazil](http://data.worldbank.org/country/brazil) | [India](http://data.worldbank.org/country/india) |
| [Indonesia](http://data.worldbank.org/country/indonesia) | [Burundi](http://data.worldbank.org/country/burundi) | [Colombia](http://data.worldbank.org/country/colombia) | [Maldives](http://data.worldbank.org/country/maldives) |
| [Kiribati](http://data.worldbank.org/country/kiribati) | [Cabo Verde](http://data.worldbank.org/country/cape-verde) | [Costa Rica](http://data.worldbank.org/country/costa-rica) | [Nepal](http://data.worldbank.org/country/nepal) |
| [Korea, Dem. Rep.](http://data.worldbank.org/country/korea-democratic-republic) | [Cameroon](http://data.worldbank.org/country/cameroon) | [Cuba](http://data.worldbank.org/country/cuba) | [Pakistan](http://data.worldbank.org/country/pakistan) |
| [Lao PDR](http://data.worldbank.org/country/lao-pdr) | [Central African Rep](http://data.worldbank.org/country/central-african-republic) | [Dominica](http://data.worldbank.org/country/dominica) | [Sri Lanka](http://data.worldbank.org/country/sri-lanka) |
| [Malaysia](http://data.worldbank.org/country/malaysia) | [Chad](http://data.worldbank.org/country/chad) | [Dominican Rep](http://data.worldbank.org/country/dominican-republic) | **Western Europe** |
| [Marshall Islands](http://data.worldbank.org/country/marshall-islands) | [Comoros](http://data.worldbank.org/country/comoros) | [Ecuador](http://data.worldbank.org/country/ecuador) | Austria |
| [Micronesia, Fed. Sts.](http://data.worldbank.org/country/micronesia-federated-states) | [Congo, Dem. Rep.](http://data.worldbank.org/country/congo-dem-rep) | [El Salvador](http://data.worldbank.org/country/el-salvador) | Belgium |
| [Mongolia](http://data.worldbank.org/country/mongolia) | [Congo, Rep.](http://data.worldbank.org/country/congo-republic) | [Grenada](http://data.worldbank.org/country/grenada) | Denmark |
| [Myanmar](http://data.worldbank.org/country/myanmar) | [Cote d'Ivoire](http://data.worldbank.org/country/cote-divoire) | [Guatemala](http://data.worldbank.org/country/guatemala) | Finland |
| [Palau](http://data.worldbank.org/country/palau) | [Eritrea](http://data.worldbank.org/country/eritrea) | [Guyana](http://data.worldbank.org/country/guyana) | France |
| [Papua New Guinea](http://data.worldbank.org/country/papua-new-guinea) | [Ethiopia](http://data.worldbank.org/country/ethiopia) | [Haiti](http://data.worldbank.org/country/haiti) | Germany |
| [Philippines](http://data.worldbank.org/country/philippines) | [Gabon](http://data.worldbank.org/country/gabon) | [Honduras](http://data.worldbank.org/country/honduras) | Greece |
| [Samoa](http://data.worldbank.org/country/samoa) | [Gambia](http://data.worldbank.org/country/gambia) | [Jamaica](http://data.worldbank.org/country/jamaica) | Iceland |
| [Solomon Islands](http://data.worldbank.org/country/solomon-islands) | [Ghana](http://data.worldbank.org/country/ghana) | [Mexico](http://data.worldbank.org/country/mexico) | Italy |
| [Thailand](http://data.worldbank.org/country/thailand) | [Guinea](http://data.worldbank.org/country/guinea) | [Nicaragua](http://data.worldbank.org/country/nicaragua) | Luxembourg |
| [Timor-Leste](http://data.worldbank.org/country/timor-leste) | [Guinea-Bissau](http://data.worldbank.org/country/guinea-bissau) | [Panama](http://data.worldbank.org/country/panama) | Netherlands |
| [Tonga](http://data.worldbank.org/country/tonga) | [Kenya](http://data.worldbank.org/country/kenya) | [Paraguay](http://data.worldbank.org/country/paraguay) | Norway |
| [Tuvalu](http://data.worldbank.org/country/tuvalu) | [Lesotho](http://data.worldbank.org/country/lesotho) | [Peru](http://data.worldbank.org/country/peru) | Portugal |
| [Vanuatu](http://data.worldbank.org/country/vanuatu) | [Liberia](http://data.worldbank.org/country/liberia) | [St. Lucia](http://data.worldbank.org/country/st-lucia) | Spain |
| [Vietnam](http://data.worldbank.org/country/vietnam) | [Madagascar](http://data.worldbank.org/country/madagascar) | [St. Vincent and the Grenadines](http://data.worldbank.org/country/st-vincent-and-the-grenadines) | Sweden |
| **Eastern Europe & Central Asia** | [Malawi](http://data.worldbank.org/country/malawi) | [Suriname](http://data.worldbank.org/country/suriname) | Switzerland |
| Albania | [Mali](http://data.worldbank.org/country/mali) | **Middle East & North Africa** | United Kingdom |
| [Armenia](http://data.worldbank.org/country/armenia) | [Mauritania](http://data.worldbank.org/country/mauritania) | [Algeria](http://data.worldbank.org/country/algeria) |  |
| [Azerbaijan](http://data.worldbank.org/country/azerbaijan) | [Mauritius](http://data.worldbank.org/country/mauritius) | [Djibouti](http://data.worldbank.org/country/djibouti) |  |
| [Belarus](http://data.worldbank.org/country/belarus) | [Mozambique](http://data.worldbank.org/country/mozambique) | [Egypt, Arab Rep.](http://data.worldbank.org/country/egypt-arab-republic) |  |
| [Bosnia and Herzegovina](http://data.worldbank.org/country/bosnia-and-herzegovina) | [Namibia](http://data.worldbank.org/country/namibia) | [Iran, Islamic Rep.](http://data.worldbank.org/country/iran-islamic-republic) |  |
| [Bulgaria](http://data.worldbank.org/country/bulgaria) | [Niger](http://data.worldbank.org/country/niger) | [Iraq](http://data.worldbank.org/country/iraq) |  |
| [Georgia](http://data.worldbank.org/country/georgia) | [Nigeria](http://data.worldbank.org/country/nigeria) | [Jordan](http://data.worldbank.org/country/jordan) |  |
| [Kazakhstan](http://data.worldbank.org/country/kazakhstan) | [Rwanda](http://data.worldbank.org/country/rwanda) | [Lebanon](http://data.worldbank.org/country/lebanon) |  |
| [Kosovo](http://data.worldbank.org/country/kosovo) | [Sao Tome and Principe](http://data.worldbank.org/country/sao-tome-and-principe) | [Libya](http://data.worldbank.org/country/libya) |  |
| [Kyrgyz Republic](http://data.worldbank.org/country/kyrgyz-republic) | [Senegal](http://data.worldbank.org/country/senegal) | [Morocco](http://data.worldbank.org/country/morocco) |  |
| [Macedonia,FYR](http://data.worldbank.org/country/macedonia-fyr) | [Sierra Leone](http://data.worldbank.org/country/sierra-leone) | [Syrian Arab Rep](http://data.worldbank.org/country/syrian-arab-republic) |  |
| [Moldova](http://data.worldbank.org/country/moldova) | [Somalia](http://data.worldbank.org/country/somalia) | [Tunisia](http://data.worldbank.org/country/tunisia) |  |
| [Montenegro](http://data.worldbank.org/country/montenegro) | [South Africa](http://data.worldbank.org/country/south-africa) | [West Bank and Gaza](http://data.worldbank.org/country/west-bank-gaza) |  |
| [Romania](http://data.worldbank.org/country/romania) | [South Sudan](http://data.worldbank.org/country/south-sudan) | [Yemen, Rep.](http://data.worldbank.org/country/yemen-republic) |  |
| [Serbia](http://data.worldbank.org/country/serbia) | [Sudan](http://data.worldbank.org/country/sudan) |  |  |
| [Tajikistan](http://data.worldbank.org/country/tajikistan) | [Swaziland](http://data.worldbank.org/country/swaziland) |  |  |
| [Turkey](http://data.worldbank.org/country/turkey) | [Tanzania](http://data.worldbank.org/country/tanzania) |  |  |
| [Turkmenistan](http://data.worldbank.org/country/turkmenistan) | [Togo](http://data.worldbank.org/country/togo) |  |  |
| [Ukraine](http://data.worldbank.org/country/ukraine) | [Uganda](http://data.worldbank.org/country/uganda) |  |  |
| [Uzbekistan](http://data.worldbank.org/country/uzbekistan) | [Zambia](http://data.worldbank.org/country/zambia) |  |  |
|  | [Zimbabwe](http://data.worldbank.org/country/zimbabwe) |  |  |

Table A2- List of ICD-9/ICD-10 Codes used to identify co-morbidities

|  | **ICD-9** | | **ICD-10** | |
| --- | --- | --- | --- | --- |
| **Problematic Alcohol** | 291  303  303.9  305.0  357.5  425.  535.3  790.3  977.3, 980,  V11.3 | Alcohol-induced mental disorders (all 291)  Alcohol dependence syndrome  Other and unspecified alcohol dependence  Nondependent abuse of alcohol  Alcoholic polyneuropathy  Alcoholic Cardiomyopathy  Alcoholic gastritis  Excessive blood level of alcohol  Poisoning by alcohol deterrents  Toxic effect of alcohol  Personal history of alcoholism | E24.4  F10  G31.2  G62.1  G72.1  I42.6  K29.2  T51  Z71.4  Z50.2 | Alcohol-induced pseudo-Cushing syndrome  Alcohol related disorders  Degeneration of nervous system due to alcohol  Alcoholic polyneuropathy  Alcoholic myopathy  Alcoholic cardiomyopathy  Alcoholic gastritis  Toxic effect of alcohol  Alcohol abuse counseling and surveillance  Alcohol Rehabilitation |
| **Alcoholic liver disease** | 571.0  571.1  571.2  571.3 | Alcoholic fatty liver  Acute alcoholic hepatitis  Alcoholic cirrhosis of liver  Alcoholic liver damage, unspecified | K70- K71 | Alcoholic liver disease  Alcoholic hepatitis  Alcoholic fibrosis and sclerosis of liver  Alcoholic cirrhosis of liver  Alcoholic hepatic failure  Alcoholic liver disease, unspecified |
| **Problematic Drug use**  **(excluding alcohol)** | 292  304  305.3-305.7  V65.42 | Drug-induced mental disorders  Drug dependence  Nondependent hallucinogen abuse/ sedative, hypnotic or anxiolytic/opioid/cocaine/amphetamine  or related acting sympathomimetic  Counseling on substance use and abuse | F11-F16  F18  F19  R78.1  R78.2  R78.3  R78.4  R78.5  Z71.5  Z72.2  Z50.3 | Opioid related disorders/Cannabis/ Sedative, hypnotic, or anxiolytic/Cocaine related/other stimulant/Hallucinogen  Inhalant related disorders  Other psychoactive substance related disorders  Finding of opiate drug in blood  Finding cocaine in blood  Finding Hallucinogen in blood  Finding other drugs of addictive potential in blood  Finding psychotropic drug in blood  Drug abuse counseling and surveillance  Drug use Excludes: abuse of non-dependence substance  and drug dependence.  Drug Rehabilitation |
| **HIV/AIDS** | 042  V08 | Human immunodeficiency virus [HIV]  Asymptomatic HIV infection status | Z21  B20-B24 | Asymptomatic HIV infection status  HIV |

Table A3- Unweighted vs. Weighted Rate of reported HCV cases per year (1998-2008)

| **Year** | **Reported rate per 100,000*** | | | | **Ratio** | |
| --- | --- | --- | --- | --- | --- | --- |
|  | **Immigrants** | | **Non-immigrants** | |  |  |
|  | **Unweighted** | **Weighted** | **Unweighted** | **Weighted** | **Unweighted** | **Weighted** |
| 1998 | 20.93 (17.54-24.31) | 28.03 (24.12-31.95) | 31.72 (30.36-33.08) | 41.14 (39.59-42.69) | 0.66 (0.56-0.78) | 0.68 (0.59-0.79) |
| 1999 | 21.34 (17.95-24.73) | 28.60 (24.67-32.52) | 33.86 (32.45-35.26) | 43.44 (41.84-45.03) | 0.63 (0.53-0.74) | 0.66 (0.57-0.76) |
| 2000 | 22.69 (19.22-26.17) | 29.40 (25.44-33.35) | 32.95 (31.56-34.33) | 41.63 (40.07-43.18) | 0.69 (0.59-0.81) | 0.71 (0.61-0.81) |
| 2001 | 28.34 (24.49-32.20) | 36.79 (32.40-41.17) | 29.21 (27.91-30.51) | 36.36 (34.91-37.82) | 0.97 (0.84-1.12) | 1.01 (0.89-1.15) |
| 2002 | 21.81 (18.49-25.13) | 27.62 (23.88-31.35) | 26.73 (25.49-27.97) | 32.80 (31.42-34.17) | 0.82 (0.70-0.96) | 0.84 (0.73-0.97) |
| 2003 | 18.52 (15.51-21.52) | 23.24 (19.88-26.61) | 24.02 (22.84-25.20) | 29.34 (28.04-30.64) | 0.77 (0.65-0.91) | 0.79 (0.68-0.92) |
| 2004 | 28.77 (25.09-32.45) | 35.54 (31.45-39.63) | 30.18 (28.87-31.50) | 36.62 (35.17-38.08) | 0.95 (0.83-1.09) | 0.97 (0.86-1.10) |
| 2005 | 28.52 (24.92-32.12) | 35.06 (31.07-39.05) | 25.09 (23.89-26.29) | 30.07 (28.76-31.39) | 1.14 (0.99-1.30) | 1.17 (1.03-1.32) |
| 2006 | 22.20 (19.07-25.32) | 27.00 (23.56-30.45) | 23.15 (22.00-24.31) | 27.51 (26.25-28.76) | 0.96 (0.83-1.11) | 0.98 (0.86-1.12) |
| 2007 | 18.91 (16.05-21.77) | 22.86 (19.72-26.01) | 19.16 (18.12-20.21) | 22.58 (21.44-23.71) | 0.99 (0.84-1.16) | 1.01 (0.87-1.17) |
| 2008 | 22.35 (17.99-26.70) | 27.06 (22.27-31.86) | 18.31 (16.87-19.75) | 21.51 (19.95-23.07) | 1.22 (0.99-1.51) | 1.26 (1.04-1.52) |
| **Overall** | 23.16 (22.13-24.20) | 29.22 (28.06-30.38) | 27.12 (26.73-27.51) | 33.48 (33.05-33.91) | 0.85 (0.81-0.90) | 0.87 (0.84-0.91) |

*Adjusted for age, sex, FSA, and year of diagnosis

**Method:** Inverse probability weighing^19,20^ was used to estimate the impact of unlinked cases [19.8% of all cases (N=5255)] on incidence rates. We imputed immigrant status (i.e. unlinked cases) with two separate models using age, sex and address based on the first 3 digits of the postal code [i.e. Forward Sorting Area (FSA)] as predictive variables for immigrant status. Using complete case characteristics, a multiple logistic regression model to predict missingness for FSA (5.8%, N=1699) as the dependent variable adjusted for age, sex, and year of diagnosis was created. The intercept and the regression coefficients for the model were used to calculate the predictive probability of missing FSA.

Given that the distribution of weights was similar for immigrants and non-immigrants a second model to predicting missingness (observed/not observed) of immigration status as the dependent variable and adjusted for age, sex, FSA, and year of diagnosis was constructed. Similarly, a weight (the inverse probability of immigration status being observed) was assigned to observed cases. A final weight was assigned as the product of weights from the two models. Weighted and unweighted incidence rates were calculated. Rate ratios with 95% CI between immigrants and non-immigrants for unweighted and weighted rates were estimated and compared.

Table A4- Demographic Characteristics of Linked and Unlinked cases (1991-2008)

| **Characteristic** | **Linked cases (n= 23,312)** | **Unlinked cases**  **(n = 5970)** | **p-value** |
| --- | --- | --- | --- |
| **Age (years)** |  |  |  |
| Mean (SD) | 43.1 (13.6) | 41.0 (14.9) | < 0.001 |
| **Age group (years)** |  |  |  |
| 0 - 4 | 86 (0.4) | 44 (0.7) | < 0.001 |
| 5 - 9 | 11 (0.05) | 9 (0.2) |  |
| 10 - 14 | 43 (0.2) | 14 (0.2) |  |
| 15 - 19 | 376 (1.6) | 148 (2.5) |  |
| 20 - 29 | 2613 (11.2) | 952 (16.0) |  |
| 30 - 39 | 6592 (28.3) | 1798 (30.1) |  |
| 40 - 49 | 7776 (33.4) | 1555 (26.1) |  |
| 50 - 59 | 3190 (13.7) | 587 (9.8) |  |
| 60 - 69 | 1328 (5.7) | 318 (5.3) |  |
| ≥ 70 | 1297 (5.6) | 355 (6.0) |  |
| Missing | - | 190 (3.2) |  |
| **Sex** |  |  |  |
| Female | 7759 (33.3) | 1970 (33.0) | 0.31 |
| Male | 15,553 (66.7) | 3826 (64.1) |  |
| Missing | - | 174 (2.9) |  |
| **Residence area by Public health region** |  |  |  |
| Montréal | 9403 (40.3) | 2942 (49.3) | < 0.001 |
| Montérégie | 3037 (13.0) | 464 (7.8) |  |
| Capitale-Nationale | 2216 (9.5) | 433 (7.3) |  |
| Laurentides | 1798 (7.7) | 539 (9.0) |  |
| Outaouais | 1094 (4.7) | 335 (5.6) |  |
| Mauricie et du Centre-du-Québec | 1165 (5.0) | 260 (4.4) |  |
| Laval | 1071 (4.6) | 294 (4.9) |  |
| Lanaudière | 951 (4.1) | 157 (2.6) |  |
| Estrie | 797 (3.4) | 157 (2.6) |  |
| Chaudière-Appalaches | 533 (2.3) | 109 (1.8) |  |
| Abitibi-Témiscamingue | 415 (1.8) | 55 (0.9) |  |
| Saguenay - Lac-Saint-Jean | 301 (1.3) | 64 (1.1) |  |
| Bas-Saint-Laurent | 205 (0.9) | 40 (0.7) |  |
| Côte-Nord | 169 (0.7) | 52 (0.9) |  |
| Gaspésie-Îles-de-la-Madeleine | 91 (0.4) | 43 (0.7) |  |
| Nord-du-Québec | 29 (0.1) | 5 (0.1) |  |
| Terres-Cries-de-la-Baie-James | 18 (0.1) | 7 (0.1) |  |
| Nunavik | 12 (0.05) | 9 (0.2) |  |
| Missing | 7 (0.03) | 5 (0.1) |  |

Table A5- Annual Frequency and Rates of all Reported HCV Cases in

the MADO database (1991-2008)

| **Year** | **Total**  **Identified cases** | **Reported rate**  **per 100,000 (95% CI)** |
| --- | --- | --- |
| **1991** | 50 | 0.7 (0.5-0.91) |
| **1992** | 78 | 1.1 (0.9-1.3) |
| **1993** | 154 | 2.2 (1.8-2.5) |
| **1994** | 219 | 3.1 (2.7- 3.5) |
| **1995** | 401 | 5.6 (5.0- 6.1) |
| **1996** | 565 | 7.8 (7.2- 8.5) |
| **1997** | 1698 | 23.5 (22.4- 24.6) |
| **1998** | 2887 | 39.8 (38.3-41.2) |
| **1999** | 3036 | 41.7 (40.2-43.2) |
| **2000** | 3039 | 41.5 (40.1-43.0) |
| **2001** | 2789 | 37.9 (36.5-39.3) |
| **2002** | 2432 | 32.9 (31.6-34.2) |
| **2003** | 2163 | 29.1 (27.8-30.3) |
| **2004** | 2630 | 35.1 (33.8-36.5) |
| **2005** | 2312 | 30.7 (29.4-31.9) |
| **2006** | 2114 | 27.9 (26.7-29.1) |
| **2007** | 1803 | 23.6 (22.5-24.7) |
| **2008** | 912 | 23.7 (22.1-25.2) |
| **Total** | **29 282** | **22.9 (22.6- 23.1)** |

Figure A1- Rates/100,000 of all reported Chronic Hepatitis C cases per year stratified by sex

Between 1998 and 2008, the age and sex adjusted HCV annual rates decreased by 5.13% per year.

The age adjusted HCV annual rates in males decreased by 5.1% per year as compared to 5.9% per year in females.
